# Differential stem cell aging kinetics in Hutchinson-Gilford progeria syndrome and Werner syndrome

**DOI:** 10.1007/s13238-018-0517-8

**Published:** 2018-02-23

**Authors:** Zeming Wu, Weiqi Zhang, Moshi Song, Wei Wang, Gang Wei, Wei Li, Jinghui Lei, Yu Huang, Yanmei Sang, Piu Chan, Chang Chen, Jing Qu, Keiichiro Suzuki, Juan Carlos Izpisua Belmonte, Guang-Hui Liu

**Affiliations:** 1 State Key Laboratory of Stem Cell and Reproductive Biology, Institute of Zoology, Chinese Academy of Sciences, Beijing, 100101 China; 2 National Laboratory of Biomacromolecules, CAS Center for Excellence in Biomacromolecules, Institute of Biophysics, Chinese Academy of Sciences, Beijing, 100101 China; 3 University of Chinese Academy of Sciences, Beijing, 100049 China; 4 National Clinical Research Center for Geriatric Disorders, Xuanwu Hospital of Capital Medical University, Beijing, 100053 China; 5 State Key Laboratory of Biomembrane and Membrane Biotechnology, Institute of Zoology, Chinese Academy of Sciences, Beijing, 100101 China; 6 Key Laboratory of Computational Biology, CAS-MPG Partner Institute for Computational Biology, Shanghai Institutes for Biological Sciences, Chinese Academy of Sciences, Shanghai, 200031 China; 7 Department of Medical genetics, School of Basic Medical Sciences, Peking University Health Science Center, Beijing, 100191 China; 8 Department of Pediatric Endocrinology and Genetic Metabolism, Beijing Children’s Hospital, Capital Medical University, National Center for Children’s Health, Beijing, 100045 China; 9 Institute for Advanced Co-Creation Studies, Osaka University, Osaka, 560-8531 Japan; 10 Graduate School of Engineering Science, Osaka University, Osaka, 560-8531 Japan; 11 Gene Expression Laboratory, Salk Institute for Biological Studies, La Jolla, 92037 USA; 12 Key Laboratory of Regenerative Medicine of Ministry of Education, Institute of Aging and Regenerative Medicine, Jinan University, Guangzhou, 510632 China

**Keywords:** WRN, lamin, HGPS, Werner syndrome, stem cell, aging

## Abstract

Hutchinson-Gilford progeria syndrome (HGPS) and Werner syndrome (WS) are two of the best characterized human progeroid syndromes. HGPS is caused by a point mutation in lamin A (*LMNA*) gene, resulting in the production of a truncated protein product—progerin. WS is caused by mutations in *WRN* gene, encoding a loss-of-function RecQ DNA helicase. Here, by gene editing we created isogenic human embryonic stem cells (ESCs) with heterozygous (G608G/+) or homozygous (G608G/G608G) *LMNA* mutation and biallelic *WRN* knockout, for modeling HGPS and WS pathogenesis, respectively. While ESCs and endothelial cells (ECs) did not present any features of premature senescence, HGPS- and WS-mesenchymal stem cells (MSCs) showed aging-associated phenotypes with different kinetics. WS-MSCs had early-onset mild premature aging phenotypes while HGPS-MSCs exhibited late-onset acute premature aging characterisitcs. Taken together, our study compares and contrasts the distinct pathologies underpinning the two premature aging disorders, and provides reliable stem-cell based models to identify new therapeutic strategies for pathological and physiological aging.

## Introduction

Progeroid syndromes are heritable human disorders characterized by progeroid features that recapitulate typical features of normal aging. Among all the progeroid syndromes, Hutchinson-Gilford progeria syndrome (HGPS) and Werner syndrome (WS) are best characterized (Kudlow et al., 2007). HGPS is a sporadic autosomal dominant syndrome, and most HGPS patients were heterozygous for *LMNA* mutation (p.G608G/+). *LMNA* encodes A-type lamins that belongs to the family of nuclear lamina proteins, and a point mutation (p.G608G) in *LMNA* creates an aberrant splicing site in exon 11, resulting in the production of a truncated protein, progerin (Chojnowski et al., 2015; DeBoy et al., 2017; Luo et al., 2014). Another commonly seen progeroid syndrome is WS, caused by mutations in *WRN* gene that encodes a RecQ DNA helicase (Yu et al., 1996) important to DNA replication and DNA damage repair. Loss-of-function WRN leads to genomic instability, heterochromatin alterations, and cell growth defects, which contribute to WS pathogenesis (Li et al., 2016; Murfuni et al., 2012; Ren et al., 2017a; Ren et al., 2011; Seki et al., 2008; Shamanna et al., 2017; Zhang et al., 2015).

Both HGPS and WS patients present a wide range of aging-associated syndromes such as alopecia, lipodystrophy, osteoporosis and atherosclerosis. Studies on fibroblasts from HGPS and WS patients reveal features of accelerated cellular senescence and decreased proliferation potential (Brunauer and Kennedy, 2015; Chen et al., 2017; Cheung et al., 2014; Cheung et al., 2015; Kudlow et al., 2007; Liu et al., 2011a). Despite these common features, differences exist between HGPS and WS in the scope, intensity and duration of symptoms. For example, most patients with HGPS show symptoms resembling aspects of aging at a very early age and die at a median age from 11 to 13. By comparison, WS patients usually develop normally in the childhood and can live up to their fifties (Cox and Faragher, 2007; Ding and Shen, 2008; Hennekam, 2006; Kudlow et al., 2007; Mazereeuw-Hautier et al., 2007; Muftuoglu et al., 2008; Oshima et al., 2017).

In recent years, technologies based on stem cells and gene editing have been widely used to model various human diseases (Atchison et al., 2017; Duan et al., 2015; Fu et al., 2016; Liu et al., 2011a; Liu et al., 2012; Liu et al., 2014; Liu et al., 2011b; Lo Cicero and Nissan, 2015; Miller et al., 2013; Pan et al., 2016; Ren et al., 2017b; Wang et al., 2017; Yang et al., 2017; Zhang et al., 2015). Of note, HGPS-specific induced pluripotent stem cells (iPSCs) and WS-specific iPSCs and embryonic stem cells (ESCs) have been separately generated. Based on the findings by us and other groups, although the iPSCs and ESCs do not have any premature aging defects, mesenchymal stem cells (MSCs) and vascular smooth muscle cells (VSMCs) derived from these pluripotent stem cells display premature aging, consistent with the observations in fibroblasts from HGPS and WS patients (Chen et al., 2017; Cheung et al., 2014; Liu et al., 2011a; Miller et al., 2013; Zhang et al., 2011). Both being typical cases of progeroid syndromes, comparative analysis on HGPS and WS is very limited. More information about the similarities and differences in the pathological processes and molecular mechanisms of HGPS and WS remains to be uncovered via comparative studies.

Here, we successfully developed a reliable and isogenic platform for side-by-side investigation of HGPS and WS. Taking advantage of gene editing, we generated human ESCs harboring heterozygous *LMNA* p.G608G mutation and *WRN* deficiency, mimicking HGPS and WS, respectively. Notably, a genetically enhanced HGPS-specific ESCs bearing biallelic *LMNA* p.G608G mutation were also created. We found that HGPS- and WS-MSCs, but not ESCs or ECs, exhibited typical aging-associated characteristics. Interestingly, distinct aging kinetics were detected between HGPS- and WS-MSCs. For the first time, we achieved a contemporaneous comparison between HGPS and WS under the same genetic background to unravel the molecular and cellular differences, opening a window into the understanding of the pathology of human aging and providing a platform for screening for therapeutic strategies against aging-associated disorders.

## Results

### Generation of *LMNA*-mutated and *WRN*-deficient human ESCs

**Figure 1:**
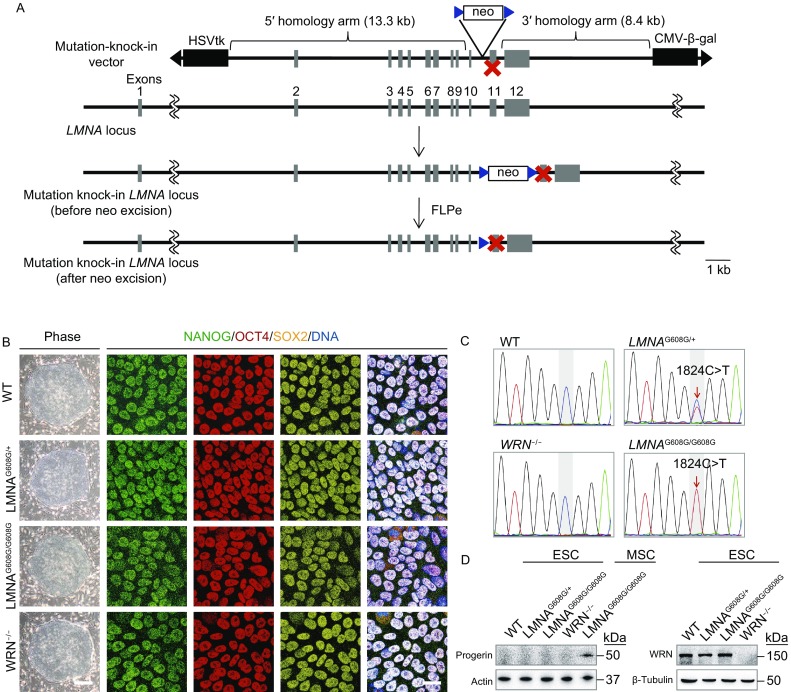
**Generation of the heterozygous (*LMNA***^**G608G/+**^**) and homozygous (*LMNA***^**G608G/G608G**^**) ESCs**. (A) Schematic representation of *LMNA* gene editing strategy by HDAdV-mediated homologous recombination. Blue triangles, *FRT* sites. (B) Morphology and immunofluorescence analysis of the pluripotency markers in WT, heterozygous (*LMNA*^G608G/+^), homozygous (*LMNA*^G608G/G608G^) and WRN^−/−^ ESCs. Scale bar, 100 μm (left); 25 μm (right). (C) Confirmation of the heterozygous and homozygous mutation of *LMNA* by DNA sequencing. (D) Immunoblotting analysis of progerin and WRN expression in WT, heterozygous (*LMNA*^G608G/+^), homozygous (*LMNA*^G608G/G608G^) and WRN^−/−^ ESCs. Progerin expression in homozygous (*LMNA*^G608G/G608G^) MSCs was carried out as a positive control

**Figure 2:**
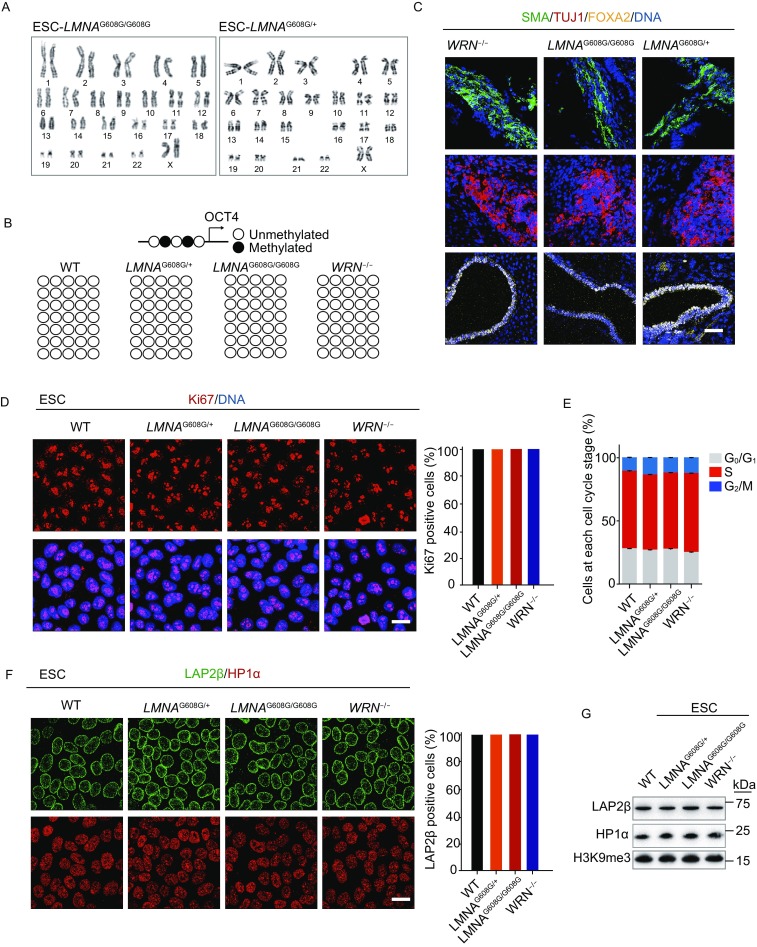
**Characterization of HGPS-ESCs and WS-ESCs**. (A) Karyotyping analysis of heterozygous (*LMNA*^G608G/+^) and homozygous (*LMNA*^G608G/G608G^) ESCs. (B) DNA methylation analysis of the *OCT4* promoter region. (C) Immunostaining of representative markers of three germ layers in teratomas derived from heterozygous (*LMNA*^G608G/+^), homozygous (*LMNA*^G608G/G608G^) and WRN^−/−^ ESCs. Scale bar, 50 μm. (D) Ki67 immunostaining analysis of WT, heterozygous (*LMNA*^G608G/+^), homozygous (*LMNA*^G608G/G608G^) and WRN^−/−^ ESCs. Scale bar, 25 μm. All cells were Ki67 positive. (E) Cell cycle analysis of ESCs. Data were presented as mean ± SEM, *n* = 3. (F) Representative immunofluorescence staining of LAP2β and HP1α in ESCs. Scale bar, 25 μm. All cells were LAP2β and HP1α positive. (G) Western blot analysis of LAP2β, HP1α and H3K9me3 expression in ESCs

Using a genome-editing technique with a helper-dependent adenoviral vector (HDAdV), we generated heterozygous and homozygous *LMNA*-mutated human ESC lines (Fig. 1A). Combined with our previously reported *WRN*-deficient human ESCs (Zhang et al., 2015), we obtained ESCs with heterozygous (*LMNA*^G608G/+^), homozygous (*LMNA*^G608G/G608G^) *LMNA* mutation, and homozygous *WRN* deficiency (*WRN*^−/−^) under the same genetic background (Fig. 1B–D). All the three ESC lines displayed normal karyotypes and morphologies indistinguishable from those of WT-ESCs (Fig. 1B and 2A). All clones expressed the pluripotency markers OCT4, SOX2, NANOG, and were hypomethylated at the *OCT4* promoter region (Fig. 1B and 2B). Each cell line was maintained for more than 30 passages without detectable growth abnormalities (data not shown) and was assessed for pluoripotency by differentiation into the three embryonic germ layers *in vivo*, by teratoma formation (Fig. 2C). Ki67 staining and cell cycle analysis also confirmed comparable proliferation potential of HGPS-ESCs and WS-ESCs with that of WT-ESCs (Fig. 2D and 2E). As expected, progerin was suppressed in both HGPS-ESCs and WS-ESCs (Fig. 1D). In addition, the levels of nuclear lamina component LAP2β, and heterochromatin markers H3K9me3 and HP1α were each normal in HGPS-ESCs and WS-ESCs compared to WT-ESCs (Fig. 2F and 2G). These data indicate that despite the progeroid-associated mutations, premature senescence phenotypes and chromosomal instability are well concealed in HGPS-ESCs and WS-ESCs at the pluripotent stage.

### HGPS-MSCs and WS-MSCs exhibit aging-associated phenotypes with different kinetics

**Figure 3:**
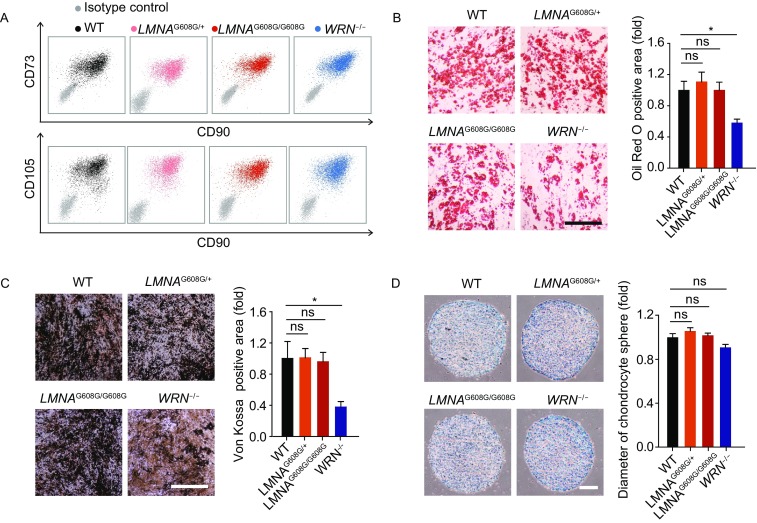
**Acquisition and characterization of HGPS-MSCs and WS-MSCs**. (A) FACS analysis of MSC-specific markers (CD73, CD90, CD105) in WT, heterozygous (*LMNA*^G608G/+^), homozygous (*LMNA*^G608G/G608G^) and WRN^−/−^ MSCs. (B) Left: characterization of adipogenesis potential of MSCs by Oil Red O staining. Right: Oil Red O positive areas were calculated by Image J. Data were presented as mean ± SEM, *n* = 3. *P < 0.05; ns, not significant. Scale bar, 100 μm. (C) Left: characterization of osteogenesis potential of MSCs by Von Kossa staining. Right: Von Kossa positive areas were calculated by Image J. Data were presented as mean ± SEM, *n* = 3. *P < 0.05; ns, not significant. Scale bar, 100 μm. (D) Left: characterization of chondrogenesis potential of MSCs by Toluidine Blue O staining. Right: the diameters of chondrocyte spheres were measured. Data were presented as mean ± SEM, *n* = 11. ns, not significant. Scale bar, 100 μm

Clinical observations in HGPS and WS patients indicate that premature aging disorders are often accompanied with defects in mesenchymal lineages, such as lipodystrophy, osteoporosis and atherosclerosis (Cox and Faragher, 2007). MSCs are adult stem cells originated from mesoderm and can be differentiated into osteocytes, chondrocytes, adipocytes and many other cell types (Lepperdinger, 2011; Marofi et al., 2017; Uccelli et al., 2008). We postulated that MSC exhaustion may play an important role in premature aging disorders. Here, HGPS-ESCs and WS-ESCs were differentiated into HGPS-MSCs and WS-MSCs. Both MSC lines expressed MSC-specific markers including CD90, CD73 and CD105 (Fig. 3A) and exhibited multiple-lineage differentiation potentials including adipogenesis, osteogenesis and chondrogenesis, though the differentiation ability of WS-MSCs towards adipocytes and osteoblasts was partly compromised (Fig. 3B–D).

**Figure 4:**
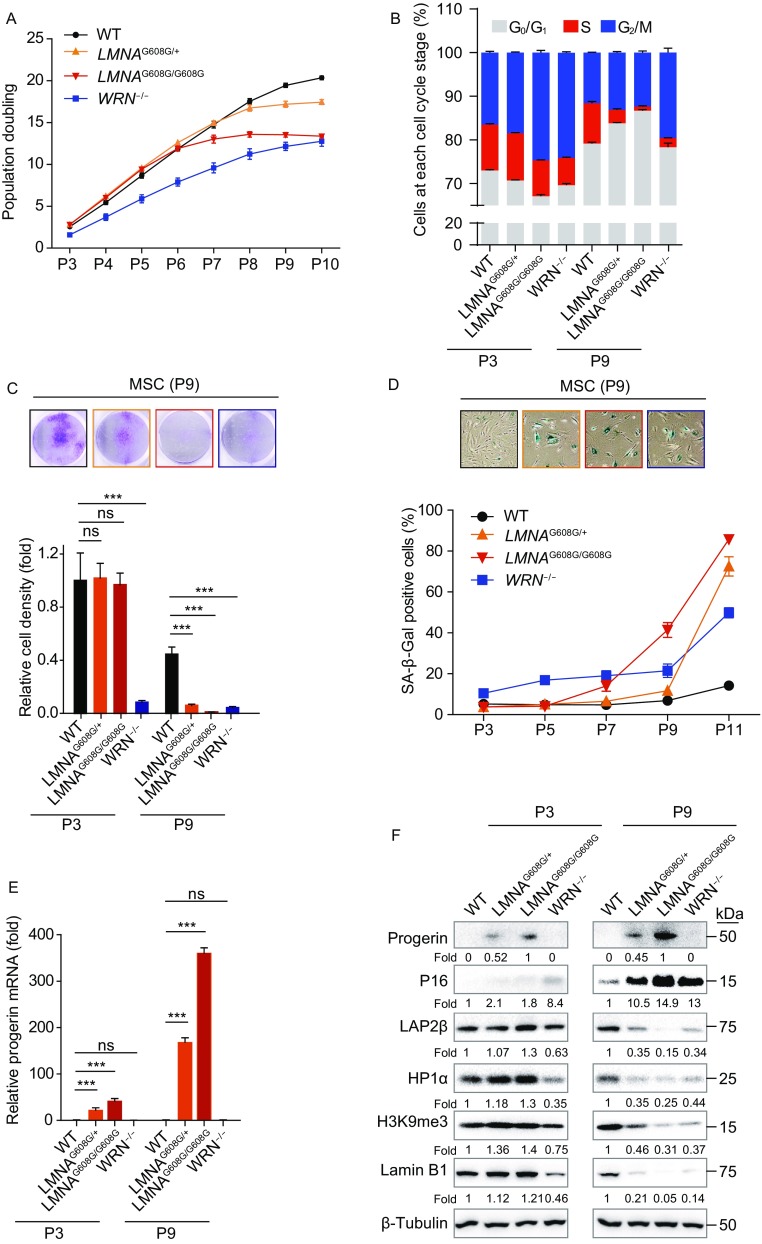
**Phenotypic analyses of HGPS-MSCs and WS-MSCs indicate different kinetics between cell models of two different progeroid syndromes**. (A) Growth curve showing the population doubling of MSCs, *n* = 3. (B) Cell cycle analysis of MSCs at passage 3 and passage 9. Data were presented as mean ± SEM, *n* = 3. (C) Analysis of clonal expansion abilities of WT, heterozygous (*LMNA*^G608G/+^), homozygous (*LMNA*^G608G/G608G^) and WRN^−/−^ MSCs. Upper: representative images of crystal violet staining at passage 9. Lower: relative clonal expansion abilities at passage 3 and passage 9. Data were shown as mean ± SEM, *n* = 3. ***P < 0.001; ns, not significant. (D) Analysis of SA-β-Gal activity in WT, heterozygous (*LMNA*^G608G/+^), homozygous (*LMNA*^G608G/G608G^) and WRN^−/−^ MSCs. Upper: representative images of SA-β-Gal staining at passage 9. Lower: frequency of SA-β-Gal positive cells. *n* = 3. (E) RT-qPCR analysis of progerin expression in WT, heterozygous (*LMNA*^G608G/+^), homozygous (*LMNA*^G608G/G608G^) and WRN^−/−^ MSCs at passage 3 and passage 9. Data were shown as mean ± SEM, *n* = 3. ***P < 0.001; ns, not significant. (F) Western blot analysis of aging-related markers in WT, heterozygous (*LMNA*^G608G/+^), homozygous (*LMNA*^G608G/G608G^) and WRN^−/−^ MSCs at passage 3 and passage 9. β-Tubulin were used as loading controls

Senescence-associated cellular changes were profiled in HGPS-MSCs and WS-MSCs at early and late passages. Population doubling curve indicated the early-onset senescence in WS-MSCs (Fig. 4A). By comparison, heterozygous (*LMNA*^G608G/+^) and homozygous (*LMNA*^G608G/G608G^) HGPS-MSCs grew at normal rate up to passage 6. Differences in cell cycle distribution were also observed between HGPS-MSCs and WS-MSCs (Fig. 4B). As previously described (Zhang et al., 2015), WS-MSCs exhibited cell cycle arrest at G_2_/M phase with decreased cell population at S phase as early as at passage 3, which later became more severe at passage 9 (Fig. 4B). By comparison, HGPS-MSCs did not show any defects until late passages, with even smaller cell population at S phase in homozygous MSCs than that in heterozygous MSCs (Fig. 4B). Consistent with the observations in growth curve and cell cycle analyses, the results of clonal expansion assay and SA-β-Gal staining also proved early-onset senescence in WS-MSCs (Fig. 4C and 4D). Interestingly, compared to the absence of progerin in HGPS-ESCs, differentiation into MSCs resulted in the re-expression of progerin at early passages and much more accumulation at late passages (Fig. 4E and 4F). With the accumulation of progerin, both heterozygous and homozygous MSCs displayed robust cell cycle arrest, proliferation defects and SA-β-Gal activity starting at passage 7, with more than 75% SA-β-Gal-positive MSCs at passage 11 (Fig. 4B–D). In addition, the doubled progerin levels (Fig. 4E–F) in homozygous HGPS-MSCs were correlated with faster kinetics of cellular senescence when compared to heterozygous HGPS-MSCs (Fig. 4A–F).

**Figure 5:**
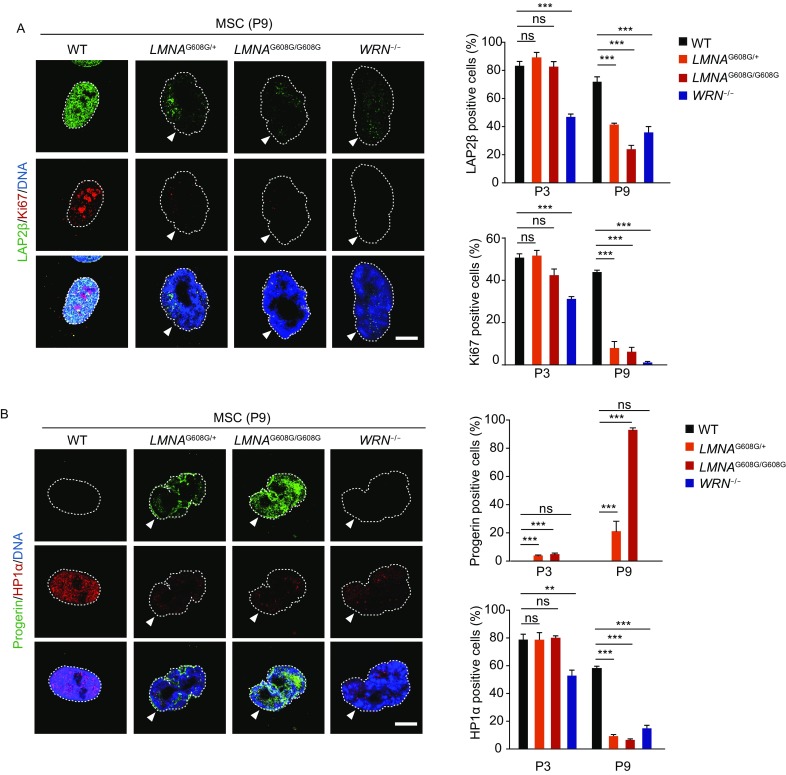
**Immunostaining of aging-related markers in HGPS-MSCs and WS-MSCs demonstrates different aging kinetics**. (A) Left: representative immunostaining of LAP2β and Ki67 in WT, heterozygous (*LMNA*^G608G/+^), homozygous (*LMNA*^G608G/G608G^) and WRN^−/−^ MSCs. Dashed lines indicate the nuclear boundaries and white arrows indicate abnormal nuclei. Scale bar, 10 μm. Right: percentages of LAP2β positive cells (upper) and Ki67 positive cells (lower) were shown as mean ± SEM, number of cells ≥ 300. ***P < 0.001; ns, not significant. (B) Left: representative immunostaining of progerin and HP1α in WT, heterozygous (*LMNA*^G608G/+^), homozygous (*LMNA*^G608G/G608G^) and WRN^−/−^ MSCs. Dashed lines indicate the nuclear boundaries and white arrows indicate abnormal nuclei. Scale bar, 10 μm. Right: percentages of progerin positive cells (upper) and HP1α positive cells (lower) were shown as mean ± SEM, number of cells ≥ 300. ***P < 0.001; **P < 0.01; ns, not significant

Consistent with the defects in cell cycle progression and clonal expansion abilities, decrease in Ki67-positive cells was accompanied by misexpression of LAP2β and decreased expression of HP1α in WS-MSCs at passage 3 and further at passage 9 (Figs. 4F, 5A and 5B), indicative of impaired proliferation potential and heterochromatin disorganization since early passages. As for HGPS-MSCs, loss of Ki67-positive cells and misexpression of LAP2β were detected only at late passages in both homozygous and heterozygous HGPS-MSCs, with a even worse LAP2β defect in homozygous HGPS-MSCs (Figs. 4F and 5A).

**Figure 6:**
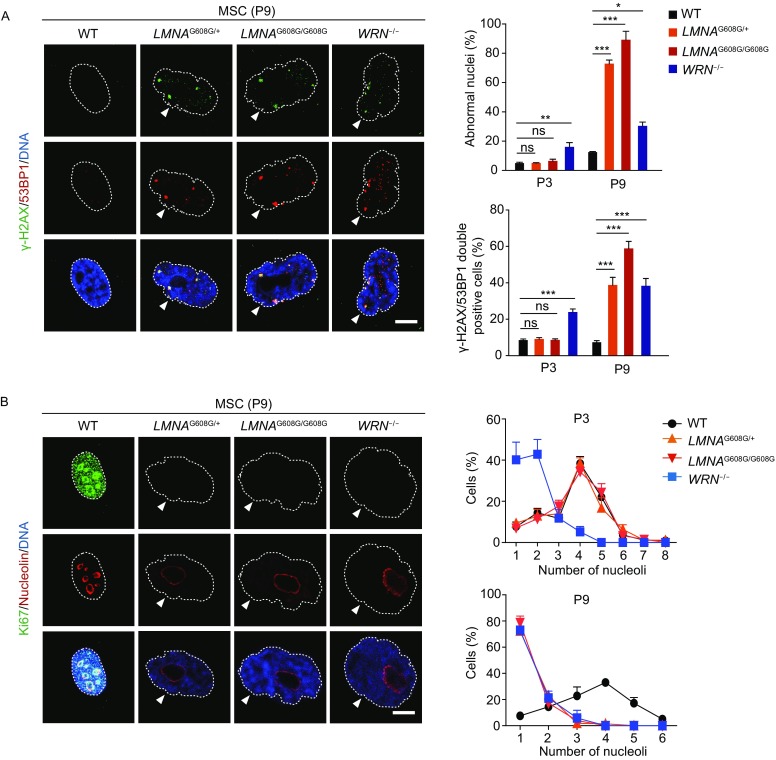
**Immunofluorescence analysis of DNA damage response and nucleolar changes in HGPS-MSCs and WS-MSCs**. (A) Left: representative immunostaining of γ-H2AX and 53BP1 in WT, heterozygous (*LMNA*^G608G/+^), homozygous (*LMNA*^G608G/G608G^) and WRN^−/−^ MSCs. Dashed lines indicate the nuclear boundaries and white arrows indicate abnormal nuclei. Scale bar, 10 μm. Right: percentages of cells with aberrant nuclear architecture (upper) and γ-H2AX/53BP1 double-positive cells (lower) were shown as mean ± SEM, number of cells ≥ 300. ***P < 0.001; **P < 0.01; *P < 0.05; ns, not significant. (B) Left: representative immunostaining of Ki67 and nucleolin in WT, heterozygous (*LMNA*^G608G/+^), homozygous (*LMNA*^G608G/G608G^) and WRN^−/−^ MSCs. Dashed lines indicate the nuclear boundaries and white arrows indicate abnormal nuclei. Scale bar, 10 μm. Right: percentages of cells with different numbers of nucleoli were shown as mean ± SEM, number of cells ≥ 300. Upper, passage 3; lower, passage 9

Previous studies have reported that cells derived from HGPS and WS patients exhibit abnormal nuclear architecture (Adelfalk et al., 2005; Choi et al., 2011; De Sandre-Giovannoli et al., 2003; Eriksson et al., 2003; Goldman et al., 2004; Mallampalli et al., 2005; Saha et al., 2014; Scaffidi and Misteli, 2006; Toth et al., 2005; Verstraeten et al., 2008; Yang et al., 2005). Here, we also observed nuclear deformations in HGPS-MSCs and WS-MSCs (Figs. 5A, 5B, 6A and 6B). Increased number of cells with abnormal nuclear architecture was seen only in WS-MSCs at passage 3, but later in both WS-MSCs and HGPS-MSCs (Fig. 6A). In fact, there were even more cells with aberrant nuclear architecture in HGPS-MSCs, especially the homozygous ones, than WS-MSCs at passage 9, correlated with increased expression levels of progerin (Figs. 4E, 4F, 5B and 6A).

Having shown the distinct senescence-associated kinetics in HGPS-MSCs and WS-MSCs, we continued to evaluate other aging-related phenotypes. Increased DNA damage response is an important feature of aging (Brunauer and Kennedy, 2015; Burtner and Kennedy, 2010; Liu et al., 2005; Lopez-Otin et al., 2013; Mostoslavsky et al., 2006; Musich and Zou, 2011; Saha et al., 2014; Wang et al., 2009; Zhang et al., 2015). Here, increase in γ-H2AX and 53BP1 double-positive cells, indicative of increased DNA damage response, was observed only in WS-MSCs at passage 3 (Fig. 6A). At passage 9, both WS-MSCs and HGPS-MSCs exhibited increased DNA damage response, with the most observed in homozygous HGPS-MSCs (Fig. 6A). Increased size and decreased number of nucleoli can also serve as aging biomarkers (Buchwalter and Hetzer, 2017; Tiku et al., 2016). We observed that only WS-MSCs had fewer but larger nucleoli at early passages, and both WS-MSCs and HGPS-MSCs exhibited increased size and decreased numbers of nucleoli at late passages (Fig. 6B).

Taken together, these results suggest that HGPS-MSCs and WS-MSCs exhibit aging-associated phenotypes with different kinetics, and progerin exerts a dose-dependent effect on cellular senescence of HGPS-MSCs.

### HGPS-ECs and WS-ECs do not exhibit phenotypes of accelerated senescence

**Figure 7:**
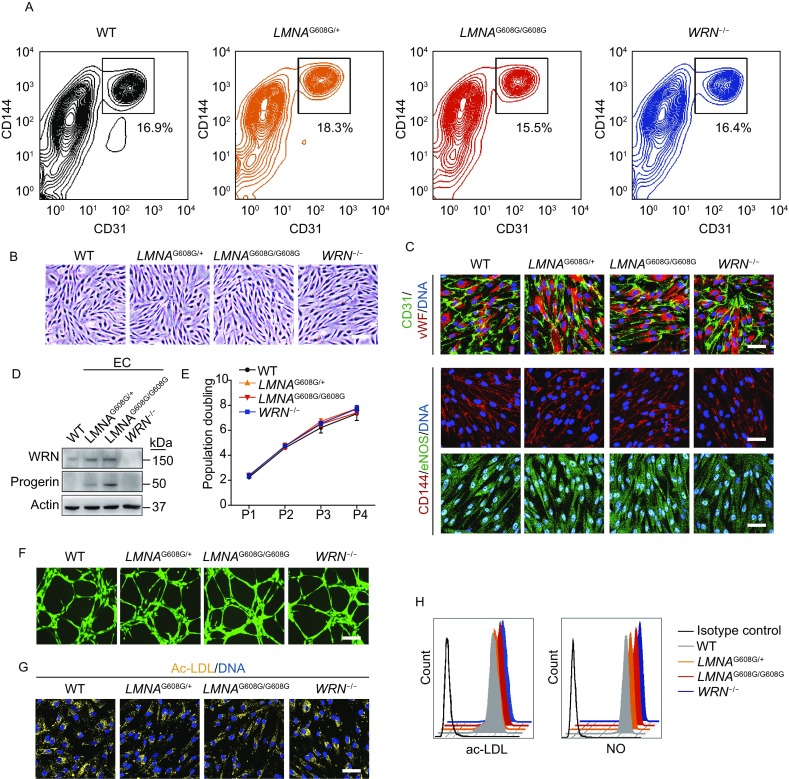
**Acquisition and characterization of HGPS-ECs and WS-ECs**. (A) CD31/CD144 positive cells were sorted as ECs by FACS. (B) Representative morphology of WT, heterozygous (*LMNA*^G608G/+^), homozygous (*LMNA*^G608G/G608G^) and WRN^−/−^ ECs. Scale bar, 50 μm. (C) Immunostaining of EC-specific markers (CD31/vWF/CD144/eNOS) in WT, heterozygous (*LMNA*^G608G/+^), homozygous (*LMNA*^G608G/G608G^) and WRN^−/−^ ECs. Scale bar, 50 μm. (D) Western blot analysis of WRN and progerin expression in WT, heterozygous (*LMNA*^G608G/+^), homozygous (*LMNA*^G608G/G608G^) and WRN^−/−^ ECs. Actin was used as loading control. (E) Growth curve analysis showing the population doubling of ECs, *n* = 3. (F) The abilities of *in vitro* tube formation in WT, heterozygous (*LMNA*^G608G/+^), homozygous (*LMNA*^G608G/G608G^) and WRN^−/−^ ECs. Cells were stained by Calcein-AM. (G) The uptake abilities of Dil-Ac-LDL in WT, heterozygous (*LMNA*^G608G/+^), homozygous (*LMNA*^G608G/G608G^) and WRN^−/−^ ECs. Scale bar, 50 μm.(H) Measurement of Dil-Ac-LDL and nitric oxide (NO) by FACS in WT, heterozygous (*LMNA*^G608G/+^), homozygous (*LMNA*^G608G/G608G^) and WRN^−/−^ ECs

**Figure 8:**
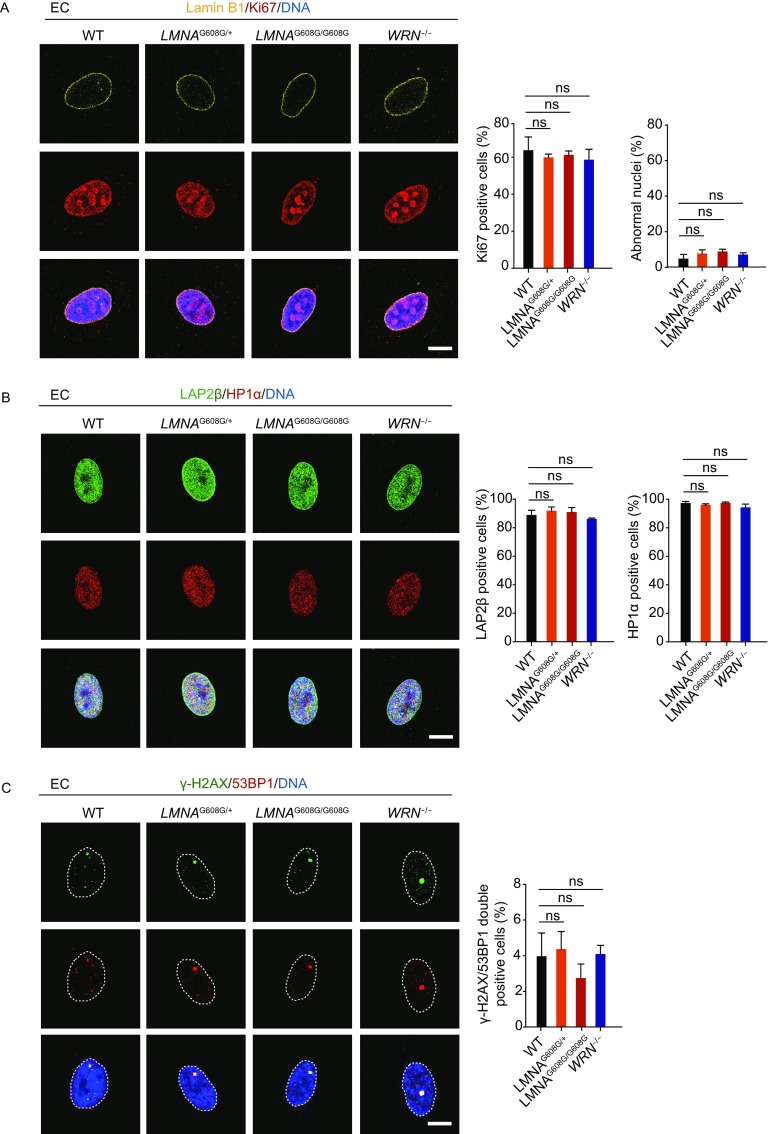
**HGPS-ECs and WS-ECs do not exhibit phenotypes of accelerated senescence**. (A) Left: representative immunostaining of Lamin B1 and Ki67 in WT, heterozygous (*LMNA*^G608G/+^), homozygous (*LMNA*^G608G/G608G^) and WRN^−/−^ ECs. Scale bar, 10 μm. Right: percentages of Ki67 positive cells and abnormal nuclei were shown as mean ± SEM, number of cells ≥ 300. ns, not significant. (B) Left: representative immunostaining of LAP2β and HP1α in WT, heterozygous (*LMNA*^G608G/+^), homozygous (*LMNA*^G608G/G608G^) and WRN^−/−^ ECs. Scale bar, 10 μm. Right: percentages of LAP2β positive cells and HP1α positive cells were shown as mean ± SEM, number of cells ≥ 300. ns, not significant. (C) Left: representative immunostaining of γ-H2AX and 53BP1 in WT, heterozygous (*LMNA*^G608G/+^), homozygous (*LMNA*^G608G/G608G^) and WRN^−/−^ ECs. Dashed lines indicate the nuclear boundaries. Scale bar, 10 μm. Right: percentages of γ-H2AX/53BP1 double-positive cells were shown as mean ± SEM, number of cells ≥ 300. ns, not significant

Arterosclerosis have been observed in HGPS and WS patients, and progerin is widely present in the vascular cells including endothelial cells (Lo et al., 2014; McClintock et al., 2006; Miyamoto et al., 2014; Olive et al., 2010). As the inner layer of blood vessels, endothelial cells have unique functions in vascular biology, including barrier effect, vascular tone control, blood clotting regulation and inflammatory response (Bochenek et al., 2016; Hansen et al., 2017; Sturtzel, 2017). To explore whether *LMNA* mutation or *WRN* deficiency may cause aging-associated defects in endothelial cells (ECs), HGPS-ESCs and WS-ESCs were differentiated into HGPS-ECs and WS-ECs, respectively. CD31 and CD144 double-positive cells were sorted (Fig. 7A). All EC lines had typical endothelial morphology (Fig. 7B) and expressed endothelial-specific markers (Fig. 7C). Despite the expression of progerin in HGPS-ECs and the loss of WRN in WS-ECs (Fig. 7D), HGPS-ECs and WS-ECs were still able to form lattice-like vessel structures on matrigel and maintain normal lipid uptake capacities, nitric oxide (NO) synthesis abilities (Fig. 7F, 7G and 7H), proliferation potentials (Fig. 7E and 8A), as well as genomic stability (Fig. 8B and 8C). Therefore, *LMNA* mutation and *WRN* deficiency does not facilitate EC senescence, suggesting that the premature aging caused by progeria-associated mutations are cell-type-specific.

**Figure 9:**
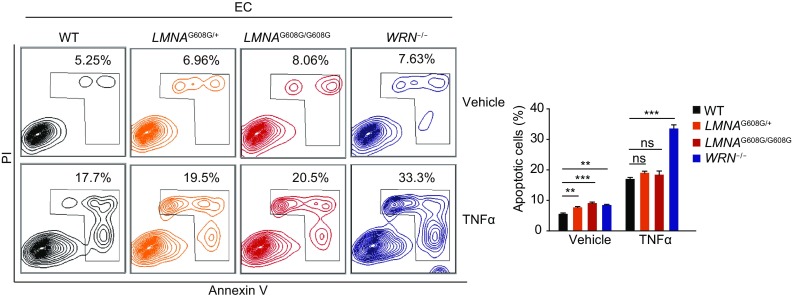
**Cellular apoptosis analysis in HGPS-ECs and WS-ECs**. Left: cellular apoptosis analysis by FACS after treatment with vehicle or TNFα in WT, heterozygous (*LMNA*^G608G/+^), homozygous (*LMNA*^G608G/G608G^) and WRN^−/−^ ECs. Right: percentages of apoptotic cells were presented as mean ± SEM, *n* = 3. ***P < 0.001; **P < 0.01; ns, not significant

To be noted, both HGPS-ECs and WS-ECs were more apoptotic compared to WT-ECs at baseline, indicating impaired EC homeostasis (Fig. 9). Additionally, WS-ECs were more sensitive to TNF-α-induced apoptosis (Fig. 9). Thus, despite the absence of premature senescence, ECs bearing HGPS or WS-associated mutations demonstrated increased susceptibility to apoptosis.

## Discussion

Human progeroid syndromes are characterized by typical features resembling normal aging, and therefore studies on progeroid disorders have provided important clues to understanding the molecular mechanisms underlying premature and normal aging (Burtner and Kennedy, 2010; Cao et al., 2011; Ding and Shen, 2008; Dreesen and Stewart, 2011; Kudlow et al., 2007; Miyamoto et al., 2014; Scaffidi and Misteli, 2006). As two of the best characterized progeroid syndromes, HGPS and WS have attracted a lot of attention during the last decade; related studies have been greatly conducive to our understanding of the pathology of these two disorders (Atchison et al., 2017; Chen et al., 2017; Cheung et al., 2014; De Sandre-Giovannoli et al., 2003; Ding and Shen, 2008; Egesipe et al., 2016; Kubben et al., 2016; Kudlow et al., 2007; Li et al., 2016; Liu et al., 2011a; Liu et al., 2011b; Lo Cicero and Nissan, 2015; Scaffidi and Misteli, 2006; Zhang et al., 2011; Zhang et al., 2015). However, there are no effective treatments so far and more information about the molecular pathology of these two premature aging syndromes are to be unveiled.

**Figure 10:**
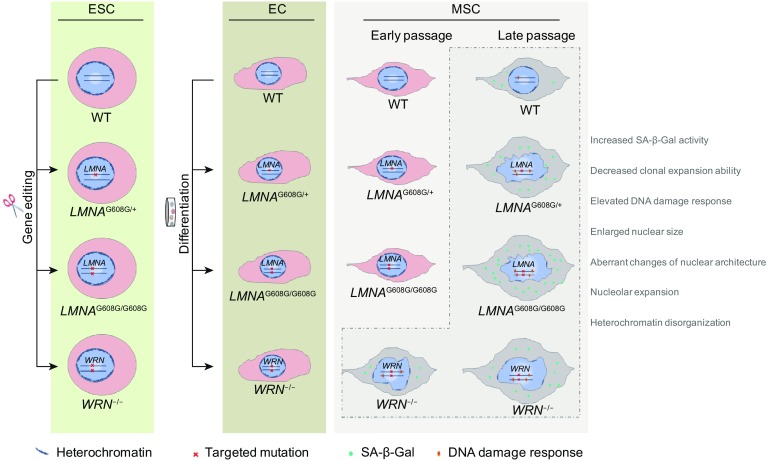
**Schematic drawing of the major cellular phenotypes observed in HGPS- and WS-specific ESCs, ECs and MSCs**. HGPS-ESCs and WS-ESCs were generated by gene editing under the same genetic background, and further differentiated to ECs and MSCs. *LMNA*-mutant and *WRN*-deficient ESCs or ECs show no accelerated senescence related defects, while HGPS-MSCs and WS-MSCs exhibited aging-associated phenotypes with different kinetics, including self-renewal ability, DNA damage response, nucleolar expansion, as well as nuclear architecture and heterochromatin alterations. WS-MSCs had early-onset mild premature aging phenotypes while HGPS-MSCs exhibited late-onset acute premature aging characterisitics

In this study, we generated *LMNA*-mutated and *WRN*-deficient human ESC lines with the same genetic background, making it possible to compare and contrast the cellular consequences of the genetic defects underlying HGPS and WS side-by-side. Similar to the iPSCs derived from the fibroblasts of HGPS and WS patients, HGPS- and WS-ESCs did not show any premature aging defects, indicating that pluripotent stem cells are able to conceal aging defects caused by *LMNA* mutation or *WRN* deficiency (Liu et al., 2012; Zhang et al., 2013). Upon mesenchymal differentiation, however, HGPS- and WS-MSCs exhibited aging-associated phenotypes that recapitulate those reported in fibroblasts and iPSC-derived MSCs from HGPS and WS patients (Cheung et al., 2014; Cheung et al., 2015; Compagnucci and Bertini, 2017; Zhang et al., 2011), with different kinetics. By measuring proliferation potential, SA-β-gal positivity, cell cycle, DNA damage response, and nuclear architecture, we showed that WS-MSCs had early-onset mild premature aging phenotypes while HGPS-MSCs exhibited late-onset acute premature aging characterisitics. To some extent, these dynamic features may mimic the patterns of disease progression of these two premature aging disorders (Fig. 10). To our knowledge, this is the first study evaluating the similarities and differences of HGPS- and WS-stem cells side by side. Our platform provides powerful tools to study aging by mimicking human genetic diseases in a petridish, facilitating the understanding of the pathology of different types of progeroid disorders and more importantly, making it possible for targeted high-throughput drug screening in human genetic background.

In addition, we observed that the homozygous HGPS-MSCs exhibited more severe aging phenotypes with a higher level of progerin than the heterozygous MSCs. Thus, the MSCs with homozygous or heterozygous *LMNA* mutation generated in our study also provide opportunities to investigate the role of progerin in a dose-dependent manner. Given the propriety of higher homogenicity in MSCs bearing homozygous *LMNA* mutation (e.g., expression of progerin), these cells may be particularly amenable to mechanistic studies using multi-omics techniques.

Different from HGPS-MSCs, HGPS-ECs did not display any premature senescence phenotypes, consistent with previous observations in HGPS-iPSC-derived ECs (Zhang et al., 2011). Similarly, WS-ECs did not show aging defects, either. These results indicate that the senescence-associated defects caused by *LMNA* mutation or *WRN* deficiency are cell-type-specific (Fig. 10). However, further analyses show that these cells were not otherwise normal; HGPS-ECs and WS-ECs were more apoptotic at baseline than WT-ECs. Moreover, WS-ECs, but not HGPS-ECs, exhibited a more pronounced response to inflammatory factor TNF-α, again indicating different molecular pathologies between the two progeroid syndromes.

MSCs and ECs as the outer and inner layers of blood vessels, respectively, play important roles in maintaining vascular homeostasis (Bochenek et al., 2016; Fang et al., 2010; Hansen et al., 2017; Hoshino et al., 2008; Kramann et al., 2016; Pasquinelli et al., 2007; Sturtzel, 2017; Wang et al., 2018). VSMCs, a cellular component of tunica media, have been proved defective in HGPS patients (Atchison et al., 2017; Chen et al., 2017; Compagnucci and Bertini, 2017; Gonzalo and Kreienkamp, 2015; Harhouri et al., 2017; Kinoshita et al., 2017; Liu et al., 2011a; Olive et al., 2010; Ragnauth et al., 2010; Vidak and Foisner, 2016; Zhang et al., 2011). Based on our data, it is reasonable to postulate that the exhaustion of MSC components in tunica adventitia may also be a common cause of accelerated aging defects in HGPS and WS patients. In addition, increased apoptosis of WS-ECs under inflammatory condition (e.g., TNF-α) may contribute to the vascular pathology in WS.

Therefore, we have generated *in vitro* models to compare and contrast the pathogenesis of HGPS and WS for the first time, providing high-throughput platforms to efficiently screen for effective treatments for both progeria syndromes and normal aging. In the future, it would be interesting to employ multi-omics technologies, including genomics, epigenomics, transcriptomics, proteomics and metabonomics, to unravel the molecular patterns of HGPS and WS under the same human genetic background, shedding light on the complex mechanisms underlying premature and normal aging and providing new evidence for the prevention and treatment of age-associated disorders.

## Materials and Methods

### Cell culture

WT-ESCs (Human H9 ESCs, WiCell Research) and three genetically edited ESCs were maintained on mitomycin C-inactivated mouse embryonic fibroblast (MEF) feeder in human ESC culture medium: 80% DMEM/F12 (Gibco), 20% Knockout Serum Replacement (Gibco), 0.1 mmol/L non-essential amino acids (NEAA, Gibco), 2 mmol/L GlutaMAX (Gibco), 55 μmol/L β-mercaptoethanol (Invitrogen), and 10 ng/mL FGF2 (Joint Protein Central); ESCs were also cultured on Matrigel (BD Biosciences) with mTeSR medium (STEMCELL Techonologies). All MSCs were cultured in MSC culture medium: 90% α-MEM + Glutmax (Gibco), 10% fetal bovine serum (FBS, Gemcell, Lot A77E01F), 1% penicillin/streptomycin (Gibco) and 1 ng/mL FGF2 (Joint Protein Central). ECs were cultured in EGM2 medium (Lonza).

### Generation of *LMNA* G608 mutation knock-in ESCs

Helper-dependent adenoviral vector (HDAdV) for *LMNA* G608G knock-in was generated same as previous report with some modifications (Yang et al., 2017). In brief, exon 11 of *LMNA* gene was PCR-amplified from *LNMA* gene correction vector (*LMNA*-c-HDAdV) (Liu et al., 2011b) and subcloned into the pCR2.1-TOPO vector (Invitrogen). The G608G mutation at exon 11 was introduced using the GeneTailor Site-Directed Mutagenesis System (Invitrogen). The mutated exon 11 was replaced into *LMNA*-c-HDAdV (Liu et al., 2011b), and generated *LMNA* G608G knock-in HDAdV plasmid. The generated plasmids was packaged into HDAdV following previous report (Yang et al., 2017). To generate heterozygous *LMNA* G608G mutation knock-in ESCs, ESCs were infected with *LMNA* G608G knock-in HDAdV at MOI of 0.3–3 btu/cell and followed previous report (Yang et al., 2017). To generate homozygous *LMNA* G608G knock-in ESCs, we repeated 2nd round of mutation knock-in using the generated heterozygous *LMNA* G608G knock-in clones. Successful targeted knock-in events were verified by PCR amplification and DNA sequencing with the following primers: *LMNA* exon 11-F, 5′-TTGGGCCTGAGTGGTCAGTC-3′; *LMNA* exon 11-R, 5′-GACCCGCCTGCAGGATTTGG-3′.

### Generation of ECs

Briefly, WT-ESCs and three genetically edited ESCs were plated on Matrigel in EC differentiation medium I (EC basal medium with 25 ng/mL BMP4, 3 μmol/L CHIR99021, 3 μmol/L IWP2 and 4 ng/mL FGF2) for 3 days. Differentiation medium II (EC basal medium with 50 ng/mL VEGF, 20 ng/mL FGF2,10 ng/mL IL-6) was used for another 3 days then CD31/CD144 double-positive cells were sorted by FACS.

### Generation of MSCs

MSCs were differentiated as previously described (Duan et al., 2015; Fu et al., 2016; Wang et al., 2018; Zhang et al., 2015). Briefly, hESCs were dissociated into EBs and then were plated on Matrigel coated plates in MSC differentiation medium (α-MEM + GlutaMAX (Gibco), 10% FBS (Gemcell, Lot A77E01F), 1% penicillin/streptomycin (Gibco), 10 ng/mL FGF2 (Joint Protein Central) and 5 ng/mL TGFβ (HumanZyme)). About 10 days later, the confluent MSC-like cells were passaged on gelatin coated plate and cultured in MSC culture medium: 90% α-MEM + Glutmax (Gibco), 10% FBS (Gemcell, Lot A77E01F), 1% penicillin/streptomycin (Gibco) and 1 ng/mL FGF2 (Joint Protein Central). Then CD73/CD90/CD105 tripositive cells were sorted by FACS. MSCs were further differentiated towards adipocytes, osteoblasts, and chondrocytes to verify their multiple-lineage differentiation capacities (Pan et al., 2016; Zhang et al., 2015). Oil red O (adiopogenesis), Von Kossa (osteogenesis), and Toluidine blue (chondrogenesis) staining was performed respectively.

### Bisulfite sequencing of the *OCT4* promoter

Bisulfite treatment of DNA was carried out by using EZ DNA Methylation Kit (Zymo Research) following the manufacturer’s instructions. About 1 μg of genomic DNA was used. A genomic fragment of the *OCT4* promoter was amplified using LA Taq Hot Start Version (TAKARA) as previously described. In brief, PCR products were purified by using gel extraction kit (Qiagen), and subsequently cloned into the pMD20 T vector (Transgen). 7 clones from each sample were sequenced with the universal primer M13.

Primers used for PCR: meF-OCT4, 5′-ATTTGTTTTTTGGGTAGTTAAAGGT-3′; meR-OCT4, 5′-CCAACTATCTTCATCTTAATAACATCC-3′.

### Teratoma analysis

Teratoma assay was performed as described (Duan et al., 2015; Fu et al., 2016; Zhang et al., 2015). Briefly, 5 × 10^6^ ESCs were administrated subcutaneously into NOD/SCID mice (male, 6–8 weeks). 6–12 weeks after injection, mice were killed and teratomas were analyzed by immunostaining. All animal experiments were conducted with approval of the Institute of Biophysics, Chinese Academy of Science.

### Fluorescence-activated cell sorting (FACS)

MSCs or ECs were collected by using TrypLE Express (Gibco) and washed by PBS twice. Cells were incubated with primary antibody diluted with 10% FBS in PBS for 1 h at room temperature and then sorted by using a flow cytometer (BD FACSAria IIIu).

Antibodies used for cell sorting: anti-CD73 (550741), anti-CD90 (555595), anti-CD31 (555445), anti-CD144 (560410) antibodies were from BD Biosciences; anti-CD105 (17–1057) antibody was from eBioscience.

### Analysis of cell cycle distribution

For cell cycle analysis, about 1 × 10^6^ cells were processed with the Click-iT EdU Flow Cytometry Assay Kits (Invitrogen) according to the manufacturer’s instructions. In brief, the cells were harvested after 2 h incubation with EdU and stained with Alexa Fluor 647 dye azide and propidium iodide. Cells were examined by fluorescence-activated cell sorting (FACS) using a flow cytometer (BD LSRFortesa).

### Clonal expansion assay

The single-cell clonal expansion assay was carried out as described (Duan et al., 2015). Briefly, 2,000 MSCs were seeded in a gelatin-coated 12-well plate. The relative cell density was then determined by Image J after crystal violet staining.

### SA-β-Gal staining

SA-β-Gal staining was performed as described previously (Duan et al., 2015; Zhang et al., 2015). Briefly, cultured cells were washed in PBS and fixed at room temperature for 5 min in 2% formaldehyde and 0.2% glutaraldehyde. Fixed cells were stained with SA-β-Gal staining solution at 37°C overnight, percentage of SA-β-Gal positive cells were then calculated.

### Measurement of cell apoptosis and nitric oxide (NO)

For cellular apoptosis analysis, cells were collected freshly and stained with Annexin V-EGFP Apoptosis Detection Kit (Vigorous Biotechnology), and then apoptotic cells were quantified by FACS. For NO detection, cells were treated with DAF-FM (Molecular Probes) for 30 min and quantified by FACS.

### Dil-Ac-LDL uptake assay

In brief, ECs were collected after 6 h incubation with Dil-Ac-LDL (Molecular Probes) in EC culture medium. For FACS analysis, cells were collected by using TrypLE Express (Gibco) and measured by a flow cytometer (BD LSRFortesa). For immunofluorescence detection, cells were processed following the immunofluorescence microscopy protocol.

### *In vitro* tube formation assay

Briefly, 5 × 10^4^ cells were suspended in 500 μL EC medium and then seeded on Matrigel coated plate. After 6–8 h, lattice-like vessel structures formed and then cells were stained with Calcein-AM (Invitrogen) and examined by using fluorescence microscope (Olympus).

### Western blotting

1 × 10^6^ cells were lysed in 100 μL RIPA buffer [50 mmol/L Tris-HCl (pH = 7.5), 150 mmol/L NaCl, 1% NP-40, 0.5% sodium deoxycholate, 0.1% SDS] supplemented with NaF, NaVO4 and a protease-inhibitor mixture (Roche). Typically 20 μg of proteins were separated by SDS-PAGE, transferred to a PVDF membrane (Millipore), and blotted with one of the following primary antibodies and then HRP-conjugated secondary antibodies. The quantification of western blot was performed with Image Lab software for ChemiDoc XRS system (Bio-Rad).

Primary antibodies for western blotting include anti-WRN (Santa Cruz Biotechnology, Inc.), anti-Progerin (Santa Cruz Biotechnology, Inc.), anti-P21 (Cell Signaling Technology, Inc.), anti-LAP2β (BD Bioscience, Inc.), anti-HP1α (Cell Signaling Technology, Inc.), anti-Actin (Santa Cruz, Inc.), anti-Lamin B1 (Abcam, Inc.), anti-P16 (BD Bioscience, Inc.), anti-H3K9me3 (Abcam, Inc.), anti-β-Tubulin (Santa Cruz, Inc.).

### RT-PCR

Total RNA was extracted by using TRIzol reagent (Invitrogen). 2 μg of RNA was converted to cDNA by using GoScript Reverse Transcription System (Promega), and 1/50 volume of the cDNA reaction was applied to PCR using primers for human GAPDH, Progerin. RT-qPCR was performed by using iTaq Universal SYBR Green Supermix (Bio-Rad).

Primers used for RT-PCR: GAPDH-F, 5′-TCGGAGTCAACGGATTTGGT-3′; GAPDH-R, 5′-TTGCCATGGGTGGAATCATA-3′; Progerin-F, 5′-ACTGCAGCAGCTCGGGG-3′; Progerin-R, 5′-TCTGGGGGCTCTGGGC-3′.

### Immunofluorescence microscopy

Cells seeded on microscope coverslips were fixed with 4% formaldehyde in PBS for 30 min, permeabilized with 0.4% Triton X-100 in PBS for 25 min, and blocked with 10% donkey serum in PBS for 1 h. The coverslips were incubated with primary antibody (diluted with 1% donkey serum in PBS) overnight at 4°C and then incubated with fluorescence-labeled secondary antibody (diluted with 1% donkey serum in PBS at 1:500) at room temperature for 1 h. Hoechst 33342 (Invitrogen) was used to stain nuclear DNA.

Antibodies for immunofluorescence were purchased from the following companies. Abcam: anti-NANOG (ab21624), anti-Nucleolin (ab22758); ZSGB-Bio: anti-hSMA (ZM-0003); Sigma: anti-TUJ1 (T2200); Santa Cruz Biotechnology: anti-OCT4 (sc-5279), anti-SOX2 (sc-17320), anti-Progerin (sc-81611), anti-Lamin B (sc-6217); Cell Signaling Technology: anti-HP1α (2616), anti-FOXA2 (8186S), anti-CD144 (2158); Bethyl Laboratories: anti-53BP1 (A300-273A); Millipore: anti-γ-H2AX (05-636); BD Bioscience: anti-LAP2β (611000), anti-eNOS (610296); Vector: anti-Ki67 (VP-RM04); Dako: anti-vWF (A082).

### Statistical analysis

Student’s *t*-test was used to analyse differences between different cell lines. Results were presented as mean ± SEM. *P* values < 0.05, *P* values < 0.01 and *P* values < 0.001 were considered statistically significant (*, **, ***).
